# Topological Reorganization of the Default Mode Network in Severe Male Obstructive Sleep Apnea

**DOI:** 10.3389/fneur.2018.00363

**Published:** 2018-06-13

**Authors:** Liting Chen, Xiaole Fan, Haijun Li, Chenglong Ye, Honghui Yu, Honghan Gong, Xianjun Zeng, Dechang Peng, Liping Yan

**Affiliations:** ^1^Department of Radiology, The First Affiliated Hospital of Nanchang University, Nanchang, Jiangxi, China; ^2^Department of General Surgery, The First Affiliated Hospital of Nanchang University, Nanchang, Jiangxi, China; ^3^Department of Cardiology, People’s Hospital of Jiangxi Province, Nanchang, Jiangxi, China

**Keywords:** obstructive sleep apnea, default mode network, cognitive function, resting-state functional magnetic resonance imaging, graph theory

## Abstract

Impaired spontaneous regional activity and altered topology of the brain network have been observed in obstructive sleep apnea (OSA). However, the mechanisms of disrupted functional connectivity (FC) and topological reorganization of the default mode network (DMN) in patients with OSA remain largely unknown. We explored whether the FC is altered within the DMN and examined topological changes occur in the DMN in patients with OSA using a graph theory analysis of resting-state functional magnetic resonance imaging data and evaluated the relationship between neuroimaging measures and clinical variables. Resting-state data were obtained from 46 male patients with untreated severe OSA and 46 male good sleepers (GSs). We specifically selected 20 DMN subregions to construct the DMN architecture. The disrupted FC and topological properties of the DMN in patients with OSA were characterized using graph theory. The OSA group showed significantly decreased FC of the anterior–posterior DMN and within the posterior DMN, and also showed increased FC within the DMN. The DMN exhibited small-world topology in both OSA and GS groups. Compared to GSs, patients with OSA showed a decreased clustering coefficient (*C*_p_) and local efficiency, and decreased nodal centralities in the left posterior cingulate cortex and dorsal medial prefrontal cortex, and increased nodal centralities in the ventral medial prefrontal cortex and the right parahippocampal cortex. Finally, the abnormal DMN FC was significantly related to *C*_p_, path length, global efficiency, and Montreal cognitive assessment score. OSA showed disrupted FC within the DMN, which may have contributed to the observed topological reorganization. These findings may provide further evidence of cognitive deficits in patients with OSA.

## Introduction

Obstructive sleep apnea (OSA) is a common sleep-disordered breathing condition characterized by repetitive cessations of breathing and/or reduced airflow due to frequent episodes of complete (apnea) or partial (hypopneas) obstruction of the upper airway during sleep. These respiratory events lead to sleep fragmentation (1), chronic intermittent hypoxemia (2), repetitive arousals, oxygen desaturation, and hypercapnic hypoxia. Moderate to severe OSA is estimated to occur in 12% of women and up to 30% of men aged between 30 and 70 years, and these estimated prevalence rates are increasing as the population ages and due to the ongoing obesity epidemic (3). OSA is associated with an increased risk of both traffic and occupational accidents (4), decreased quality of life, and long-term health problems resulting from a number of concomitant diseases, including hypertension, cardiovascular impairment, stroke, chronic kidney disease (5), depression (6), anxiety, metabolic syndrome, insomnia, cognitive dysfunction, and even Alzheimer’s disease (7). OSA is also associated with cognitive dysfunction, which is an important independent predictor of mortality, even in the absence of dementia manifestations. Cognitive deficits, including deficits in attention, memory, psychomotor function, executive functions, visuospatial function, and language ability, have been observed in patients with OSA (8, 9). Unfortunately, the neurological basis of neurocognitive dysfunction in patients with OSA has not been examined in detail.

Neuroimaging studies have been widely applied to explain these cognitive deficits and have revealed that patients with OSA show alterations in multiple brain regions, which are responsible for cognitive, affective, autonomic, and sensorimotor control (10–13). According to recent resting-state functional magnetic resonance imaging (rs-fMRI) studies, patients with OSA exhibited significant global and regional connectivity deficits, particularly in the default mode network (DMN) (14), salience network (SN), central executive network (CEN) (15).

The DMN is critical for maintaining brain function in the resting-state and experiences progressive deactivation as the brain engages in goal-directed activity. The DMN is a large-scale network that includes a set of highly interconnected brain regions, such as the posterior cingulate cortex (PCC), precuneus, medial prefrontal cortex, and the medial, lateral and inferior parietal regions, which contribute to internal mentation, attention, and adaptive functions (16). In previous studies, patients with OSA showed significant regional deficits in spontaneous activity in DMN subregions (17–19). In addition, Zhang found patients with OSA exhibited structural and functional deficits in the anterior DMN and functional compensation in the posterior DMN (20) using independent component analysis (ICA). Moreover, Li et al. observed altered functional connectivity (FC) between eight pairs of DMN subregions, which was associated with cognitive impairment (21). Patients with OSA show abnormal deactivation in the DMN during working memory tasks. The deactivation of DMN regions is significantly associated with behavioral performance and episodic memory impairments, plays a role in cognitive impairment in patients with OSA (14). However, these previous studies were limited to the spontaneous abnormalities in local brain regions and did not directly assess important topological changes in the DMN of patients with OSA.

Accumulating evidence implicates aberrant activity in the DMN in cognitive impairments and symptoms associated with neuropsychiatric disorders, such as mild cognitive impairment (22), social anxiety disorder (23), primary insomnia (24), and depression (25). Functional alterations in the DMN have been proposed as a quantitative MRI assessment that may facilitate the clinical prognosis and diagnosis (26). Previous study that utilized graph theory approaches revealed alterations in the topological properties of the gray matter volume (GMV) structural network (27) and the brain functional network (28) in individuals with OSA. However, whether the FC is altered within the subregions of the DMN and the topological changes that occur in the DMN in patients with OSA remain unclear.

Here, we hypothesized that the cognitive impairment observed in patients with OSA might be attributed to disrupted FC and the topological configuration of the DMN, and the topological reorganization may probably related to abnormal DMN FC. To test our hypothesis, we applied graph theory approaches to analyze FC and the topological organization of the DMN in male patients with untreated severe OSA and examined the relationships between neuroimaging measures and clinical index.

## Materials and Methods

### Participants

Fifty male patients with newly diagnosed untreated severe OSA and 46 male education- and age-matched good sleepers (GSs) were recruited from the Sleep Monitoring Room of the Respiratory Department at the First Affiliated Hospital of Nanchang University, China, from June 2015 to February 2017. Sex differences, depression, obesity, and anxiety may affect spontaneous brain activity, and female OSA patients exhibited a lower apnea–hypopnea index (AHI), which was frequently accompanied by depression and anxiety (29–32). To improve the credibility of our study, we only recruited untreated male patients with severe OSA to rule out potential confounders of sex differences, severity of OSA, depression, and anxiety. The inclusion criteria for patients with OSA and GSs were (1) OSA: an AHI greater than or equal to 30; GSs: an AHI less than 5; (2) male sex; (3) right-handedness; and (4) aged older than 20 years but less than 60 years. The exclusion criteria for all participants were (1) a history of other sleep disorders, such as insomnia or sleep-related eating disorder; (2) identifiable focal or diffuse abnormalities in structural MR images; (3) a history of neurological or mental illnesses (e.g., head injury, depression, psychosis, neurodegenerative diseases, hypothyroidism, and epilepsy); (4) a history of addiction; (5) a history of cerebrovascular disease; and (6) MRI contraindications, such as claustrophobia, metallic implants, or devices in the body. The study protocol was approved by the Medical Research Ethics Committee and the Institutional Review Board of the First Affiliated Hospital of Nanchang University. The current study was conducted according to the principles of the Declaration of Helsinki and the approved guidelines. Written informed consent was obtained from all participants.

### Overnight Polysomnography (PSG)

Prior to collecting MRI brain scans, overnight PSG was performed on all participants using the Respironics LE-Series Physiological Monitoring System (Alice5 LE, FL, USA) to confirm the OSA/GS diagnosis and to exclude other sleep disorders. On the day prior to overnight PSG, all participants were required to refrain from using hypnotics and consuming alcoholic beverages or coffee. Overnight PSG was recorded from 10:00 p.m. to 6 a.m. A standard electroencephalogram (EEG, F4/M1, C4/M1, O2/M1, F3/M2, C3/M2, and O1/M2), chin electromyogram, electrocardiogram, electrooculogram, thoracic and abdominal respiratory movements, oral and nasal airflow, oxygen saturation (SaO2), body posture, and snoring were recorded. Studies were scored by a PSG technician and reviewed by a qualified sleep medicine physician according to the American Academy of Sleep Medicine (AASM) guidelines (33). Obstructive apnea was defined as any 10 s or longer decrease in airflow ≥90% with evidence of persistent respiratory effort. Hypopnea was defined as a reduction in airflow ≥30% lasting for more than 10 s, accompanied by 4% or greater oxygen desaturation and/or EEG arousal (33). The AHI was computed as the mean number of apnea and hypopnea events per hour during sleep. The arousal index (AI) was calculated as the average number of EEG arousals per hour of sleep.

### Neuropsychological Assessments

Each participant was evaluated with the Epworth sleepiness scale (ESS) (Chinese version) for excessive daytime sleepiness, which requires the participant to rate his/her probability of falling asleep in eight different situations on a scale of increasing probability from 0 to 3. The aggregate score of the ESS is 24, with a score greater than 6 indicating sleepiness, a score greater than 11 indicating excessive sleepiness, and a score greater than 16 suggesting risky sleepiness. In addition, we used the Montreal Cognitive Assessment (MoCA, Chinese version) (34) as a rapid screening tool to assess cognitive function in all participants, including executive function, calculation, memory, attention, abstraction, language, and orientation. The total MoCA score is 30, with a score less than or equal to 26 indicating the presence of a mild cognitive impairment.

### MRI Data Acquisition

All MRI data were collected on a 3.0-T MRI system (Siemens, Erlangen, Germany) by implementing an 8-channel phased-array head coil at the First Affiliated Hospital of Nanchang University, China. Comfortable fixed foam pads were used to reduce head movements and ear plugs were used to minimize scanner noise. First, each participant underwent conventional T1 and T2-weighted imaging to exclude the presence of massive brain lesions. Then, both an 8-min rs-fMRI scan with an echo planar imaging sequence [repetition time (TR) = 2,000 ms, echo time (TE) = 30 ms, field of view (FOV) = 230 mm × 230 mm, thickness = 4.0 mm, gap = 1.2 mm, flip angle = 90°, matrix = 64 × 64, slices = 30] and high-resolution three-dimensional T1-weighted structural MR images using a magnetization-prepared rapidly acquired gradient echo sequence with generalized autocalibrating partially parallel acquisition (GRAPPA) for K space fill (TR = 1,900 ms, TE = 2.26 ms, FOV = 250 mm × 250 mm, thickness = 1.0 mm, gap = 0.5 mm, flip angle = 9°, resolution matrix = 256 × 256, slices = 176) were collected. During the rs-fMRI scan, all subjects were asked to remain motionless, relax, keep their eyes closed, and avoid thinking systematically or falling asleep. After the MRI scan, the participants were asked whether they fell asleep and/or avoided thinking systematically during the entire scan.

### Functional Magnetic Resonance Imaging Data Preprocessing

Image preprocessing was performed using the Data Processing & Analysis Assistant for Resting-State Brain Imaging (DPABI, Chinese Academy of Sciences, Beijing, China^1^) (35) and Statistical Parametric Mapping (SPM8),^2^ which is run on the MATLAB R2012a (MathWorks, Natick, MA, USA) platform. Preprocessing included the following steps: (1) the first 10 volumes of each functional time series were discarded; (2) slice timing correction was performed for the remaining 230 volumes; (3) three-dimensional head motion correction was conducted for small head movements; (4) high-resolution T1-weighted structural images were co-registered to the mean realigned functional images for each individual, and the transformed T1 structural images were segmented into gray matter, white matter, and cerebrospinal fluid using a new segment algorithm with the diffeomorphic anatomical registration through exponentiated lie algebra (DARTEL) tool (36), the realigned functional volumes were spatially normalized to the Montreal Neurological Institute (MNI) space using the normalization parameters estimated in DARTEL, and then each voxel was re-sampled to 3 mm × 3 mm × 3 mm; (5) the images were spatially smoothed with a 6-mm full-width at half-maximum Gaussian kernel; (6) the time series were further linearly detrended, and temporal bandpass filtering (0.01–0.08 Hz) was performed to reduce the effect of physiological high-frequency noise and low-frequency drifts; and (7) to further reduce possible sources of artifacts, the nuisance signal (white matter, cerebrospinal fluid, and global signal) and the Friston 24-parameter model (37) were regressed from the time series of all voxels using multiple regression analyses. The participants were excluded if the maximum head motion of maximum rotation was more than 2.0°, the maximum orthogonal direction displacement was more than 2.0 mm, or the mean relative root mean square was greater than 0.2 mm, according to the criteria (38, 39). Four patients with OSA were excluded. Finally, 46 male patients with untreated severe OSA and 46 male age- and education-matched GSs were included in the current study.

### DMN Construction and Graph Analyses

#### Definition of DMN Subregions

According to a previous study, we focused on the DMN and chose a specific set of 20 regions of interest (ROI) with substantial agreement with the functional and anatomic partitions of the DMN (Table 1) (16).

**Table 1:** Regions of interest within the default mode network (DMN).

Regions	Abbreviation	Brodmann areas	Montreal Neurological Institute (MNI)		
			*x*	*y*	*z*
Anterior medial prefrontal cortex	aMPFC.L	10, 32	−6	52	−2
	aMPFC.R		6	52	−2

Posterior cingulate cortex	PCC.L	23, 31	−8	−56	26
	PCC.R		8	−56	26

Dorsal medial prefrontal cortex	dMPFC	9, 32	0	52	26

Temporal parietal junction	TPJ.L	40, 39	−54	−54	28
	TPJ.R		54	−54	28

Lateral temporal cortex	LTC.L	21, 22	−60	−24	−18
	LTC.R		60	−24	−18

Temporal pole	TempP.L	21	−50	14	−40
	TempP.R		50	14	−40

Ventral medial prefrontal cortex	Vmpfc	11, 24, 25, 32	0	26	−18

Posterior inferior parietal lobule	pIPL.L	39	−44	−74	32
	pIPL.R		44	−74	32

Retrosplenial cortex	Rsp.L	29, 30, 19	−14	−52	8
	Rsp.R		14	−52	8

Parahippocampal cortex	PHC.L	20, 36, 19	−28	−40	−12
	PHC.R		28	−40	−12

Hippocampal formation	HF.L	20, 36	−22	−20	−26
	HF.R		22	−20	−26

*Coordinates are based on the MNI coordinate system, and each region of the DMN was acquired by spherical seeds with a radius of 6 mm*.

#### DMN Functional Connectivity and Graph Analyses

A network is composed of a set of nodes and edges between different nodes. The mean time series for each voxel within the ROI of the DMN was extracted using spherical seeds (6 mm in radius) based on the MNI coordinate system. Next, the Pearson correlation coefficients were computed between each pair of DMN subregions in each participant to generate a 20 × 20 correlation matrix of the DMN. Then, we used the graph theoretical network analysis (GRETNA)^3^ toolbox (40) to evaluate the topological organization of the DMN.

### Threshold Selection

In this study, the DMN was modeled based on an undirected, binarized method. The establishment of a sparsity threshold (Sp), which is defined as the fraction of the number of existing edges divided by the maximum possible number of edges in a network, ensured that the resulting networks had the same number of edges and minimized the influence of potential confounders on the overall correlation strength between groups (41). In the present study, we computed the network properties of the DMN over a wide range of sparsity levels (from 0.05 to 0.50 using an interval of 0.01), in which the number of spurious edges was minimized and the small-world parameters could be properly estimated (42).

### Network Metrics

In this study, we used the graph theory approach to calculate the global and nodal network properties of the DMN in patients with OSA and GSs. The area under the curve (AUC) of each network metric was calculated for statistical comparison, which was extracted by thresholding across a range of sparsity values to depict changes in the topological characterization of brain networks, and which is susceptible to detecting topological alterations of brain disorders (41, 43).

### Global Network Metrics

#### Small-World Parameters

**Figure 1:**
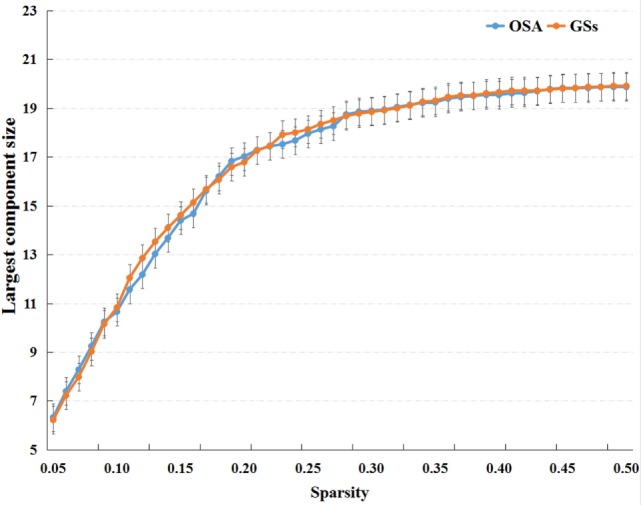
The largest component sizes of individual networks both of the patients with obstructive sleep apnea (OSA) and good sleepers (GSs) over the sparsity range of 0.05–0.50.

Small-world parameters (1) small-worldness, σ, is a fascinating model for the description of complex brain networks that not only support both specialized and integrated information processing but also facilitates an energy-efficient balance between network segregation and integration. Mathematically, a real brain network is considered a small-world network if it displays a much higher clustering coefficient (*C*_p_) and a similar characteristic path length (*L*_p_) (compared with 1,000 matched random networks in our study) and meets the following criteria: normalized clustering coefficients $\gamma =C_{{\text{p}}_{\text{real}}}/C_{{\text{p}}_{\text{rand}}}>1$ and normalized characteristic path length $\lambda =L_{{\text{p}}_{\text{real}}}/L_{{\text{p}}_{\text{rand}}}\approx 1$. The small-worldness, σ = γ/λ, is typically >1 for small-world networks (44, 45); (2) The clustering coefficient of node *i* (*Ci*) is defined as the percentage of the number of existing connections among the node’s nearest neighbors and the maximum possible number of connections. The clustering coefficient of network *C*_p_ is the average of *Ci* across nodes, which is a measure of network segregation (44); (3) The characteristic path length, *L*_p_, is quantified as the average of the shortest path length that links all pairs of nodes in the network, which is the most commonly used measure of network information integration (45). The characteristic path length was calculated as the “harmonic mean” distance between all possible pairs of regions to deal with the possible disconnected graphs dilemma in the present study (46). The largest component sizes of individual networks over the sparsity range of 0.05–0.50 with an interval of 0.01, see in Figure 1.

#### Network Efficiency

Network efficiency, including global efficiency, *E*_glob_, which represents the capacity of parallel information transmission over the network, and local efficiency, *E*_loc_, represents the capacity of a network to transmit information at the local level and measures the fault tolerance of the network (42).

### Regional Network Metrics

The degree for a brain region is defined as the number of edges of a node that connect with the remaining nodes in the network, thus measuring how interactive a particular node is in the network. The nodal betweenness is designated as the fraction of shortest paths between two nodes passing through the area in the network and measures the influence of a region on network communication. Nodal efficiency is defined as the inverse of the harmonic mean of the shortest path length in the network, quantifying the importance of the nodes for communication within the network (42).

### Statistical Analysis

Demographic and clinical characteristics of the OSA and GS groups were compared using independent two-sample *t*-tests with IBM Statistical Package for the Social Sciences 20.0 software (IBM SPSS Inc., Chicago, IL, USA). Independent two-sample *t*-tests were performed to compare group differences in the AUC of global network metrics and ROI-ROI FC of the DMN. We also compared nodal properties between patients with OSA and GSs and Bonferroni correction was performed for multiple comparison. The effects of age, body mass index (BMI), and educational level were diminished by a regression analysis. Abnormal DMN FC was calculated as the average of the correlation coefficients of the DMN in patients with OSA that showed significant between-group differences. The relationships between abnormal DMN FC and topological metrics of the DMN, and the relationships between network metrics with significant between-group differences and clinical indices in the OSA group were investigated using a Pearson correlation analysis. *p* < 0.05 was considered statistically significant.

## Results

### Demographic and Clinical Data

As shown in Table 2, significant inter-group differences were observed in BMI, AHI, total sleep time, Stage 1, rapid eye movement (REM), AI, SaO_2_ < 90%, average SaO2, oxygen desaturation index, nadir SaO2, MoCA score, visuospatial/executive, delayed memory, attention, abstraction, orientation and ESS score (*p* < 0.05). No inter-group differences in Stage 2 or Stages 3 + 4 were observed (*p* > 0.05).

**Table 2:** Comparison of the demographic and clinical data from the patients with OSA and GSs.

Characteristics	Patients with OSA (*N* = 46)	GSs (*N* = 46)	*t*-Value	*p*-Value
BMI, kg/m^2^	27.52 ± 3.30	23.09 ± 1.96	7.827	<0.001*
AHI/h	58.26 ± 20.37	2.51 ± 1.21	18.529	<0.001*
Total sleep time, min	372.26 ± 83.88	398.30 ± 18.94	−2.054	0.043*
Stage 1, %	31.28 ± 17.38	10.22 ± 3.72	8.037	<0.001*
Stage 2, %	39.12 ± 14.78	40.74 ± 7.05	−0.672	0.504
Stages 3 + 4, %	22.49 ± 18.21	21.15 ± 4.54	0.483	0.630
REM, %	7.29 ± 7.96	21.89 ± 7.48	−9.070	<0.001*
Arousal index/h	40.36 ± 23.63	11.93 ± 2.79	8.102	<0.001*
SaO_2_ < 90, %	31.15 ± 21.34	0.27 ± 0.17	9.813	<0.001*
Average SaO_2_, %	90.69 ± 4.46	95.59 ± 2.41	−6.547	<0.001*
Oxygen desaturation index	54.42 ± 25.51	2.84 ± 1.4	14.897	<0.001*
Nadir SaO_2_, %	66.26 ± 12.46	90.33 ± 2.88	−12.765	<0.001*
MoCA score	25.17 ± 2.11	27.74 ± 1.39	−6.883	<0.001*
Visuospatial/executive	4.07 ± 0.83	4.67 ± 0.63	−3.960	<0.001*
Delayed memory	3.20 ± 1.17	4.85 ± 0.36	−9.172	<0.001*
Attention	5.33 ± 0.99	5.83 ± 0.38	−3.194	0.002*
Language	2.04 ± 0.56	2.83 ± 0.38	−7.860	<0.001*
Abstraction	1.50 ± 0.51	1.85 ± 0.36	−3.790	<0.001*
Orientation	5.72 ± 0.66	5.93 ± 0.25	−2.102	0.038*
ESS score	12.11 ± 3.84	3.39 ± 2.18	13.405	<0.001*

*Data are presented as the mean ± SD*.

*OSA, obstructive sleep apnea; GSs, good sleepers; BMI, body mass index; AHI, apnea–hypopnea index; REM, rapid eye movement; SaO_2_ < 90%, percentage of total sleep time spent at an oxygen saturation less than 90%; MoCA, Montreal cognitive assessment; ESS, Epworth sleepiness scale; N, number*.

*p < 0.05, which was considered statistically significant.

### Changes in FC Within the DMN Between Patients With OSA and GSs

**Figure 2:**
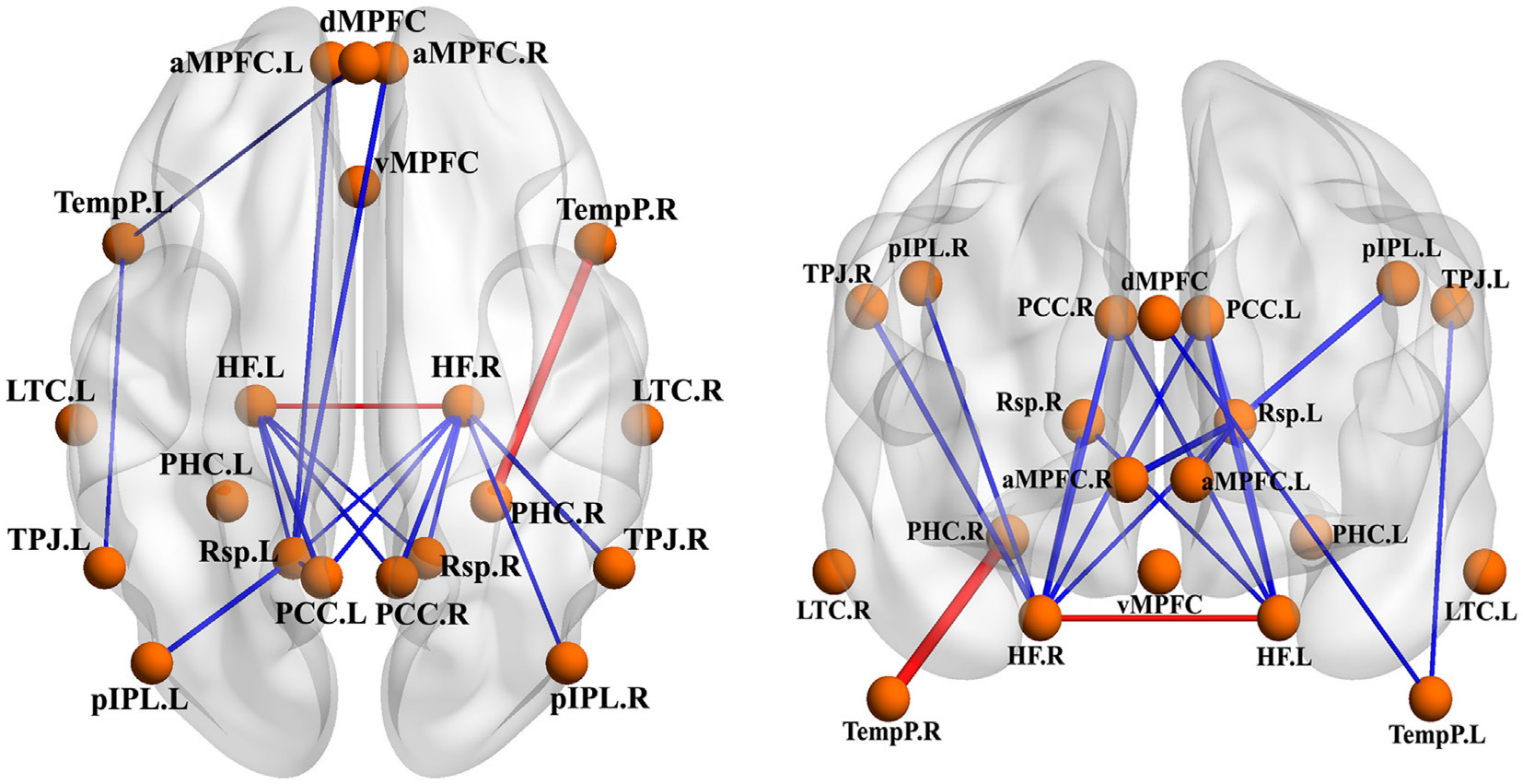
Abnormal functional connectivity (FC) within the default mode network (DMN) between patients with obstructive sleep apnea (OSA) and good sleepers (GSs). The blue edges represent decreased FC in patients with OSA compared to GSs and the red edges represent increased FC within the DMN. Undirected edges correspond to *t*-values, with a larger *t*-value corresponding to a thicker edge (*p* < 0.05, uncorrected).

**Figure 3:**
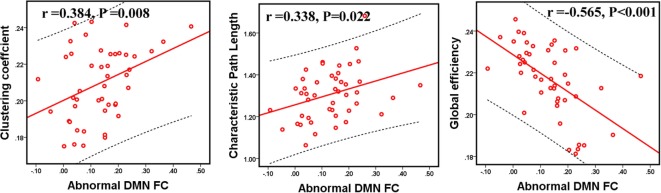
The relationship between abnormal functional connectivity (FC) and topological metrics of the default mode network (DMN) in patients with obstructive sleep apnea (OSA). The abnormal DMN FC value was significantly correlated with *C*_p_, *L*_p_, and *E*_glob_ in patients with OSA. *p* < 0.05, which was considered statistically significant.

Compared to GSs, patients with OSA exhibited significantly decreased FC between the bilateral PCC and the bilateral hippocampal formation (HF) and left retrosplenial cortex (Rsp), between the left temporal pole (TempP) and the dorsal medial prefrontal cortex (dMPFC) and left temporal parietal junction (TPJ), between the left Rsp and the bilateral anterior medial prefrontal cortex (aMPFC) and the left posterior inferior parietal lobule (pIPL), between the bilateral HF and the bilateral Rsp, and between the right HF and the right TPJ and the right pIPL. Patients with OSA displayed significantly increased FC in the DMN between the right TempP and the right parahippocampal cortex (PHC) and between the right and left HF, compared to GSs (Figure 2; Table 3). The abnormal DMN FC was positively correlated with *C*_p_ (*r* = 0.384, *p* = 0.008) and *L*_p_ (*r* = 0.338, *p* = 0.022), and negatively correlated with *E*_glob_ (*r* = −0.565, *p* < 0.001) in patients with OSA (see Figure 3).

**Table 3:** Abnormal functional connectivity (FC) within the default mode network (DMN) between patients with obstructive sleep apnea (OSA) and good sleepers (GSs).

Brain region 1	Brain region 2	*t-*Value	*p*-Value
PCC.L	Rsp.L	−2.490	0.015
PCC.L	HF.L	−2.948	0.004
PCC.L	HF.R	−2.479	0.015
PCC.R	HF.L	−2.412	0.018
PCC.R	HF.R	−2.940	0.004
TempP.L	dMPFC	−2.146	0.035
TempP.L	TPJ.L	−2.137	0.035
TempP.R	PHC.R	2.100	0.039
Rsp.L	aMPFC.L	−2.555	0.012
Rsp.L	aMPFC.R	−3.086	0.003
Rsp.L	pIPL.L	−3.045	0.003
HF.L	Rsp.L	−2.257	0.026
HF.L	Rsp.R	−2.050	0.043
HF.L	HF.R	2.557	0.012
HF.R	TPJ.R	−2.791	0.006
HF.R	pIPL.R	−2.417	0.018
HF.R	Rsp.L	−2.096	0.039
HF.R	Rsp.R	−2.555	0.012

*Abnormal FC within the DMN between patients with OSA and GSs (*p* < 0.05, uncorrected)*.

*L, left; R, right; PCC, posterior cingulate cortex; Rsp, retrosplenial cortex; HF, hippocampal formation; TempP, temporal pole; dMPFC, dorsal medial prefrontal cortex; TPJ, temporal parietal junction; PHC, parahippocampal cortex; aMPFC, anterior medial prefrontal cortex; pIPL, posterior inferior parietal lobule*.

### Differences in Global Network Measures of the DMN

**Figure 4:**
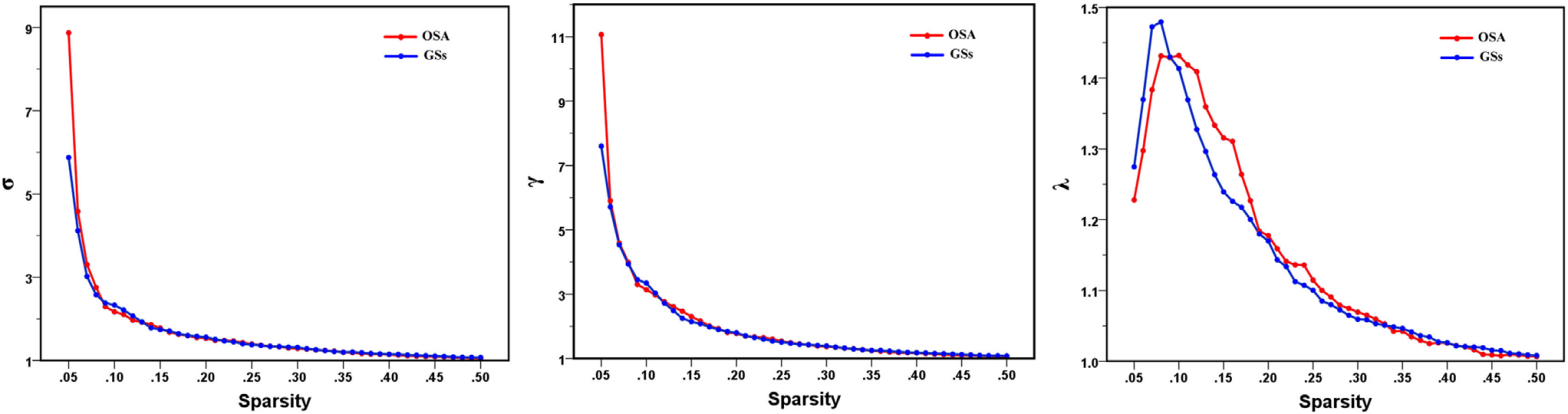
Small-world parameters of default mode network in patients with obstructive sleep apnea (OSA) and good sleepers (GSs). Graphs show that in the defined wide range of thresholds, both the patients with OSA and GSs exhibited normalized clustering coefficient (γ) obviously larger than 1, normalized path lengths (λ) approximately equal to 1, and small-worldness (σ) larger than 1, suggesting that both OSA patients and GSs show typical small-world topology.

**Figure 5:**
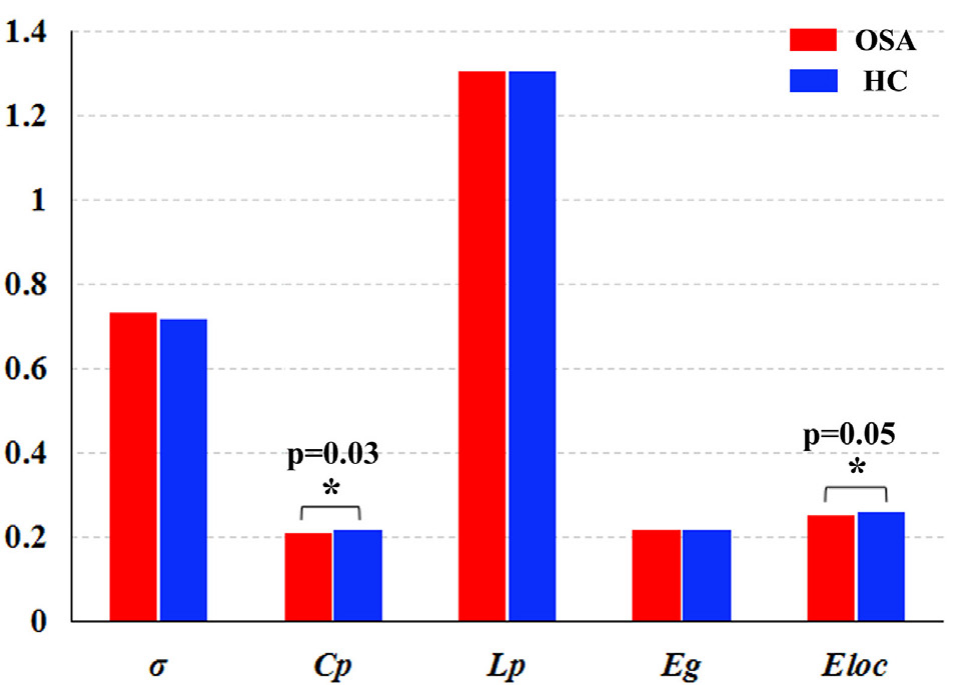
Graphs showing the small-world parameters and network efficiency of the default mode network in patients with obstructive sleep apnea (OSA) and good sleepers (GSs). Although OSA and GSs have typical small-world topology, compared to GSs, patients with OSA showed a significantly decreased *C*_p_ (*t* = −2.200, *p* = 0.030) (*p* < 0.05, uncorrected), and a decreased *E*_loc_ (*t* = −1.943, *p* = 0.054), which have a trend for difference.

In the defined wide range of thresholds (here from 0.05 to 0.50), both the patients with OSA and GSs exhibited σ value larger than 1, γ value obviously larger than 1, and λ value of approximately equal to 1 (see Figure 4), suggesting that both patients with OSA and GSs have typical small-world topology. However, compared to GSs, patients with OSA showed a significantly decreased *C*_p_ (*t* = −2.200, *p* = 0.030) and a decreased *E*_loc_ (*t* = −1.942, *p* = 0.054), which have a trend for difference. There was no significant difference in σ (*t* = 0.412, *p* = 0.483), *L*_p_ (*t* = −0.004, *p* = 0.997) or *E*_glob_ (*t* = −0.035, *p* = 0.972). Global network measures are illustrated in Figure 5.

### Group Differences in Regional Network Measures of the DMN

Patients with OSA showed abnormal nodal centrality, which showed significant between-group differences in at least one nodal metric, including nodal betweenness, nodal efficiency, and nodal degree. Compared with the GSs, patients with OSA showed decreased nodal centralities in the left PCC and dMPFC, and increased nodal centralities in the vMPFC and the right PHC (*p* < 0.05, uncorrected). Regional network measures are illustrated in Table 4.

**Table 4:** Between-group differences in regional network measures of the default mode network (DMN) in patients with obstructive sleep apnea (OSA) and good sleepers.

DMN region	Nodal betweenness		Nodal degree		Nodal efficiency	
	*t*-Value	*p*-Value	*t*-Value	*p*-Value	*t*-Value	*p*-Value
PCC.L	−4.427	<0.001*	−2.883	**0.005^#^**	−3.552	0.001*
dMPFC	−1.989	**0.049^#^**	−1.375	0.172	−1.324	0.189
vMPFC	2.475	**0.015^#^**	1.172	0.244	0.403	0.688
PHC.R	2.017	**0.047^#^**	2.833	**0.006^#^**	2.074	**0.041^#^**

*Patients with OSA showed abnormal nodal centrality in PCC.L, dMPFC, vMPFC, and PHC.R, which showed significant between-group differences in at least one of the three nodal metrics*.

*^#^p < 0.05, uncorrected*.

*Bonferroni correction p = 0.05.

*PCC.L, left posterior cingulate cortex; dMPFC, dorsal medial prefrontal cortex; vMPFC, ventral medial prefrontal cortex; PHC.R, right parahippocampal cortex*.

### Correlations Between Network Measures With Group Differences and Clinical Variables

**Figure 6:**
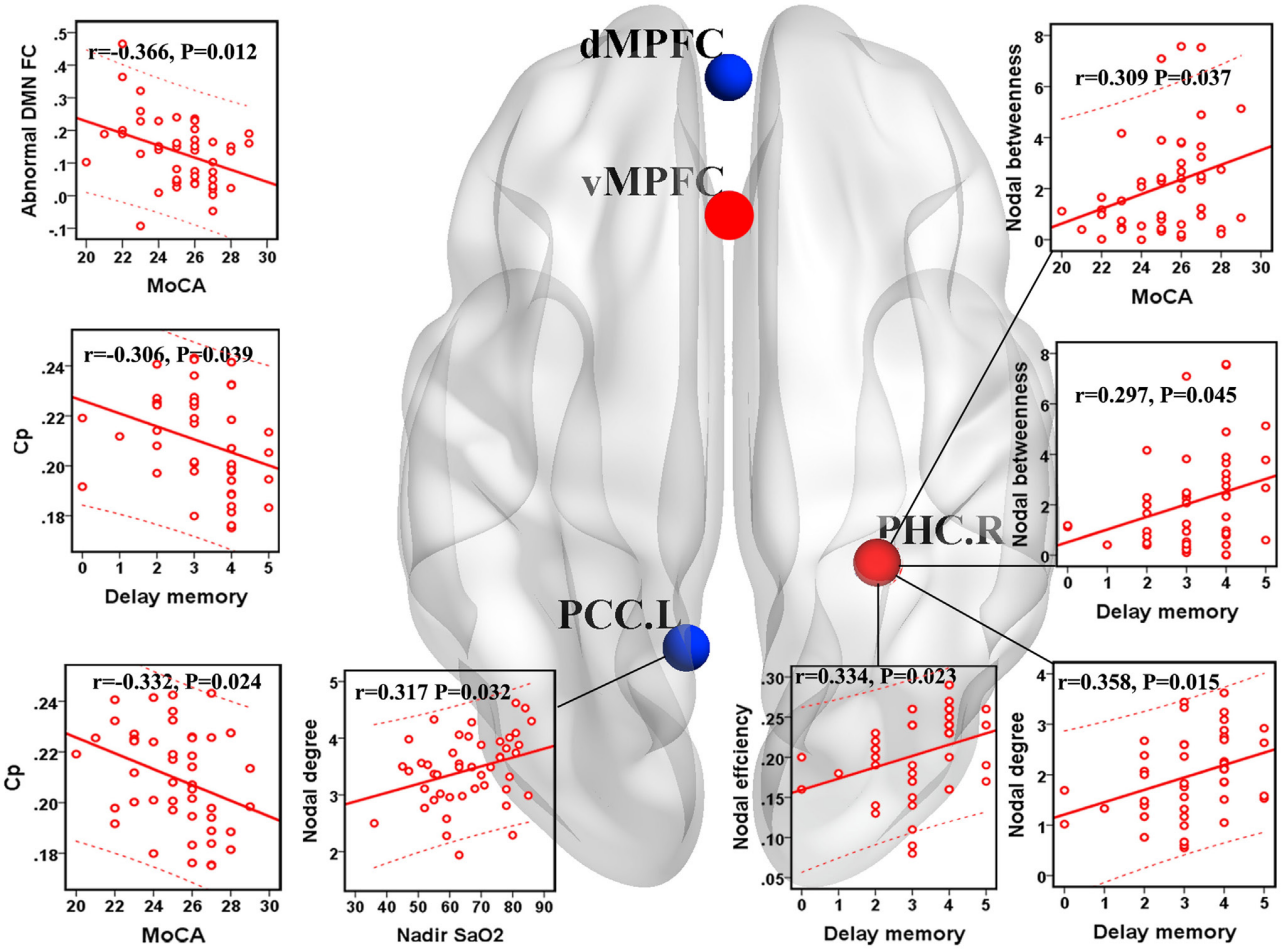
Correlations between network measures with group differences and clinical variables in patients with obstructive sleep apnea (OSA). Scatter plot showed the relationship between the aberrant network attribute parameters and clinical index in patients with OSA. The red ball represents the increased nodal centrality and the blue ball represents the decreased nodal centrality. *E*_loc_, local efficiency; *C*_p_, clustering coefficient; PCC.L, left posterior cingulate cortex; PHC.R, right parahippocampal cortex.

Within the OSA group, the abnormal DMN FC was negatively correlated with the MoCA score (*r* = −0.366, *p* = 0.012). *C*_p_ was negatively correlated with the MoCA score (*r* = −0.332, *p* = 0.024) and delayed memory (*r* = −0.306, *p* = 0.039). The nodal degree of the left PCC was positively correlated with the nadir SaO_2_ (*r* = 0.317, *p* = 0.032), and nodal betweenness of the right PHC was positively correlated with the MoCA score (*r* = 0.309, *p* = 0.037). The nodal betweenness (*r* = 0.297, *p* = 0.045), degree (*r* = 0.358, *p* = 0.015), and efficiency (*r* = 0.334, *p* = 0.023) of the right PHC were positively correlated with delayed memory (see Figure 6).

## Discussion

The present study applied graph theory approaches to provide evidence that the cognitive impairments observed in patients with OSA might be attributed to the topological configuration of the DMN, which probably resulted from the abnormal DMN FC. Although the DMN of patients with OSA exhibited small-world properties, patients with OSA showed decreased *C*_p_ and *E*_loc_, abnormal nodal centralities in the DMN, and abnormal FC within the DMN, implying a disturbance in the functional differentiation of the DMN. In addition, the abnormal DMN FC was related to *C*_p_, *L*_p_, *E*_glob_, and the MoCA score. The disrupted topological properties of the DMN significantly influenced cognitive function, including delayed memory and memory extraction in patients with OSA.

### Abnormal FC Within the DMN in Patients With OSA

The current study revealed significantly decreased FC in the anterior–posterior DMN involving the prefrontal, parietal and temporal regions in patients with OSA. Zhang et al. (20) found that patients with OSA exhibited decreased FC in the anterior DMN and a compensatory increased FC in the posterior DMN. Decreased FC in the anterior–posterior DMN indicated that the transmission of information and integration of long-distance connectivity between different regions may be damaged in patients with OSA.

We also observed significantly decreased FC in the posterior DMN, which includes the PCC, HF, temporal, and parietal lobes and the limbic system. *E*_loc_ predominantly reflects short-distance connections between neighboring regions (45). Decreased short-distance connections that are primarily located in the posterior DMN may lead to decreased *C*_p_ and decreased *E*_loc_ of the DMN in patients with OSA. The PCC and HF are connected anatomically and functionally, and these functional interactions are presumed to underlie normal episodic memory capacity (47). Patients with OSA showed decreased FC between the right HF and the PCC, which is related to delayed memory (21). Based on the results, the OSA group showed decreased FC between the bilateral PCC and the bilateral HF, consistent with previous studies (21). Decreased FC in the anterior–posterior DMN and posterior DMN may further indicate cognitive impairments in patients with OSA (48).

Park observed abnormal FC in various brain regions, and altered FC subsequently resulted in disrupted topological properties in patients with OSA, particularly in the integrative aspects of brain network organization (49). Given the significant association between abnormal DMN FC and *C*_p_, *L*_p_, and *E*_glob_ of the DMN in the current study, we believe that disrupted FC within the DMN may contribute to the topological reconfiguration of the DMN in patients with OSA. Furthermore, abnormal DMN FC was associated with the MoCA score. Therefore, the abnormal DMN FC may partially explain the impaired cognitive function and topological reconfiguration in patients with OSA.

### Global Network Measures of the DMN

Patients with OSA have recently been shown to display an abnormal small-world organization in both functional (28, 49) and structural (27) brain networks. In the present study, both patients with OSA and GSs showed efficient economic small-world organization in the DMN. Although the DMN has small-world properties, our results identified decreased *C*_p_ and *E*_loc_ of the DMN in patients with OSA. Thus, individuals with OSA likely have sparse connectivity and disconnections between adjacent brain regions in the DMN, resulting in decreased *C*_p_ and *E*_loc_. *C*_p_ is a metric that quantifies the strength of network segregation (50). The present results indicate a decline in functional differentiation in the DMN, suggesting that highly local specialization and the integrity of the DMN may be impaired in patients with OSA. *E*_loc_ essentially reflects the fault tolerance of the network and the capacity for transmitting information over local networks (45). Our finding of a decreased *E*_loc_ suggests disrupted DMN architecture in patients with OSA that is characterized by higher vulnerability and a decreased capacity for regional information processing. Moreover, *C*_p_ was negatively correlated with the MoCA score and delayed memory, further illustrating that disrupted global topology of the DMN influence cognitive impairments in patients with OSA, including delayed memory and memory extraction.

### Regional Network Measures

Nodal betweenness centrality, nodal efficiency, and nodal degree were combined to compare the regional topological organization between patients with OSA and GSs in our study. Deceased nodal centrality was identified in the PCC and dMPFC. The PCC has strong reciprocal connections with other structures involved in cognitive function (51), the collection and evaluation of information, attention processing, personal significance, and evoked emotion (16). Previous structural neuroimaging studies have observed decreased GMV (52) and white matter integrity (53) in the PCC in patients with OSA. Furthermore, patients with OSA show decreased brain activation, decreased degree centrality, and FC alterations in the PCC (17, 18, 20, 21, 54). DMN dysfunction is associated with impairments in cognitive performance (55, 56). Intermittent hypoxia is a major factor in DMN dysfunction in patients with OSA (14, 21). We also observed a positive correlation between the nodal degree of the left PCC and nadir SaO2, suggesting that the functional damage of the PCC was related to intermittent hypoxia, which may be a major factor involved in DMN dysfunction and may further explain cognitive dysfunction in patients with OSA.

The dMPFC subsystem includes the dMPFC, temporal parietal junction, lateral temporal cortex, and TempP, which are involved in social cognition, metacognition, and mental state inference (16). Patients with OSA displayed decreased FC and reduced GMV in the MPFC of the anterior DMN, indicating structural and functional deficits (20). The OSA group showed decreased nodal centrality of the dMPFC, but a compensatory increase in nodal centrality of the ventral medial prefrontal cortex in the present study, which may also confirm the deficiency in the dMPFC subsystem of the DMN in patients with OSA.

The PHC plays an important role in episodic memory, autobiographical memory and episodic simulation, spatial memory, scene perception, and spatial navigation (47). Previous voxel-based morphometry studies revealed that atrophy (57) and regional cerebral blood flow were significantly reduced in the bilateral PHC (58), which may be related to cognitive impairments in patients with OSA. Nevertheless, we found a compensatory increase in nodal centrality in the right PHC, which was positively correlated with delayed memory, may partially explain the deficits in memory, spatial learning, memory extraction and attention in patients with OSA.

### Limitations

Several limitations in this study should be addressed. First, we only revealed the small-world properties of the DMN, but patients with OSA exhibited disruptions in the DMN, as well as the SN and CEN (15). Therefore, further investigations of other specific brain networks are necessary. Second, we only specifically selected 20 nodes of the DMN (16) and characterized the DMN using an unbiased seed-based FC approach. More nodes of the DMN should be used to construct the DMN and the present findings should be validated by ICA. Third, the global network measures, nodal centrality, and ROI-ROI FC results were not corrected by multiple comparisons, meaning that this study should be considered an exploratory analysis. In addition, a more detailed neuropsychological assessment questionnaire must be used to obtain more interesting data.

## Conclusion

In the current GRAPPA study, patients with OSA showed disrupted FC and topological reorganization of the DMN. Abnormal DMN FC may contribute to the topological configuration of the DMN and cognitive impairment in patients with OSA. These results provide important insights into the neurobiological mechanisms of both disrupted FC and disrupted network properties of the DMN, which may partially account for the impaired cognitive function in patients with OSA.

## Ethics Statement

The study protocol was approved by the Medical Research Ethics Committee and the Institutional Review Board of the First Affiliated Hospital of Nanchang University. The current study was conducted according to the principles of the Declaration of Helsinki and the approved guidelines. Written informed consent was obtained from all participants.

## Author Contributions

All authors listed have made a substantial, direct, and intellectual contribution to the work and approved it for publication.

## Conflict of Interest Statement

The authors declare that the research was conducted in the absence of any commercial or financial relationships that could be construed as a potential conflict of interest.

## Footnotes

^1^http://www.restfmri.net (Accessed: 2008).

^2^http://www.fil.ion.ucl.ac.uk (Accessed: 1994).

^3^http://www.nitrc.org/projects/gretna/ (Accessed: December 12, 2012).
